# Determinants of High-tech Exports: New Evidence from OECD Countries

**DOI:** 10.1007/s13132-023-01116-z

**Published:** 2023-02-08

**Authors:** Amadeo Navarro Zapata, María Arrazola, José de Hevia

**Affiliations:** Economic Analysis Department, Universidad Rey Juan Carlos, Madrid, Spain

**Keywords:** Manufacturing trade, Technological intensity, Factor endowment, Trade, Industrial policies

## Abstract

High export capacity is a key element for sustained long-term economic growth. To achieve this goal, the technological sophistication of exports plays a key role. To enhance exports with a high level of technological sophistication, it is critical to target key drivers of high-tech exports. Hence, this article studies the determinants of international trade flows of manufactures according to their technological content in the case of OECD countries. Given a panel of 35 countries and 15 years (2004 to 2018), panel data estimation techniques are used in the analysis. In addition, two alternative measures have been considered to measure the importance of high-technology content manufacturing exports: High-tech manufacturing exports and high-tech manufacturing exports as a share of total employment. Results obtained show strong evidence of the relevance of variables such as gross fixed capital formation on total employment, the land area per capita, the percentage of university graduates relative to the population group, R&D expenditure in terms of GDP, the stock of inward foreign direct investment in terms of GDP, imports of high-tech manufactures as a share of GDP, the quality of national governance and regulation, the country population and EU membership as determinants of technology-intensive exports. Moreover, the findings have significant implications for trade and industrial policies in OECD countries, to ensure the effectiveness of policies targeting the technological upgrading of exports.

## Introduction

In many countries exports are an important contributor to GDP as well as a crucial factor in pricing efficiency and allocation of scarce resources to the most export-intensive sectors. Therefore, many governments have implemented policies and other measures to increase foreign demand for domestic products. Traditionally, it was assumed that the export structure of countries depended on relative factor endowments, which led to more developed countries specialising in the production of more technology-intensive goods, while less developed countries specialised in more resource-intensive and labour-intensive goods. More recently, new theories of international trade have focused the study on issues such as the existence of economies of scale, economies of agglomeration, factor linked to increasing returns to scale, externalities and catching-up as determinants of trade patterns. Therefore, trade policies have focused on improving competitive capabilities by targeting potential determinants of export performance, such as skill formation, technology upgrading, fixed capital formation, foreign direct investment and strategic and institutional factors among others (Krugman, 1986), to enhance the creation of dynamic comparative advantages. In recent years, authors such as Palley (2008) have tried to overcome the criticisms of the traditional concept of comparative advantage made by scholars such as Krugman (1979), whereas factors such as innovation play an important role in determining dynamic comparative advantages.

The analysis of export capacity has been a subject of eager interest over the last decades (Warner & Kreinin, 1983; Moreno, 1997; Egger, 2001; Jongwanich, 2010). However, although more attention has recently been paid to the technological component of manufacturing exports, largely because of its important implications for economic growth (Crespo & Wörz, 2005; Falk, 2009; Jarreau & Poncet, 2012; Nouira & Saafi, 2022) and productivity (Ekananda & Parlinggoman, 2017), research on the determinants of manufacturing exports is still scarce. Therefore, it is important to analyse the determinants that lead some countries to export more manufactures with higher technological content than others, to pursue economic policies that encourage these drivers and thus increase the technological content of manufacturing exports.

In this context, this research examines drivers of technology-intensive manufacturing exports from OECD countries. The sample period considered is between 2004 and 2018. Therefore, a panel of 35 countries and 15 years is considered. Thus, the article covers a broad period, which includes a major event in the world economy, such as the Great Recession in 2009. In contrast to the empirical literature in this area, which focuses on a few countries, highly heterogenous countries in terms of their levels of development or over-concentrated in geographic areas, this article covers all OECD countries, which have certain standards of homogeneity in terms of economic development, the same vision on functioning mechanisms within the market economy and with limited geographic concentration. Moreover, OECD countries are considered by many developing countries as role models when implementing economic policies to upgrade economic development performance. Thus, the analysis of the drivers of technology-intensive manufacturing exports may also help developing countries in export promotion policy design.

In addition to this introduction, the article covers five other sections. The “Literature Review” section reviews the literature on the analysis of the drivers of technology-intensive exports. The “Data and Methodology” section describes the data and methodology used. The “Results” section shows the results obtained in the empirical analysis, which are discussed in the “Discussion” section. Subsequently, the article presents the most important conclusions of the study.

## Literature Review

The authors such as Hausman et al. (2007), Rodrik (2006) and Minondo (2010) have shown the need to incorporate export sophistication and diversification into the analysis of exports, as long-term economic growth hinges not exclusively on the volume of exports but notably on its technological intensity. Previously, the authors such as Granstrand (1998) showed through microeconomic analysis that firms with a strong technological base tend to economise on costly new technologies and achieve this through the implementation of internationalisation strategies, diversification of technology-related businesses, commercialisation and external supply of technology, rationalisation of R&D and strategic partnerships related to technology. More recently, Ekananda and Parlinggoman (2017) studied the role of high-tech exports and foreign direct investment on economic growth, concluding that exports of high-tech goods positively affect GDP through productivity.

Given the relevance of the technological intensity of exports as a tool in achieving economic growth, a large empirical literature has been developed that analyses the determinants of the technological intensity of exports. One of the earliest studies in the analysis of the drivers of technology-intensive exports is the research carried out by Seyoum (2004), which uses data from 54 geographically dispersed countries to analyse the role of variables such as human capital, technology, domestic demand, competition, exchange rate and foreign investment on the technological level of countries’ exports. Braunerhjelm and Thulin (2008) analyse the high-tech exports of 19 OECD countries between 1981 and 1999 and find evidence of the positive role of R&D expenditure in higher technology content manufacturing exports and in determining comparative advantage across countries. Tebaldi (2011) based on data from a wide range of economically and geographically highly heterogenous countries examines high-tech exports drivers between 1980 and 2008, finding evidence that the quality of human capital, foreign direct investment and countries’ openness to international trade are key factors in the export performance of countries exporting high-tech products. Gökmen and Turen (2013) study the determinants of high-tech exports in fifteen EU countries concluding that foreign direct investment, the level of human development and the level of economic freedom play an important role as drivers of high-tech exports. Also, Basarac et al. (2013) studied the drivers of high-tech manufacturing exports from EU countries, concluding that domestic demand and industrial production play an important role in high-tech manufacturing exports. Still for the EU countries, Sandu and Ciocanel (2014) studied the impact of research and development and innovation on high-tech export, results confirmed a positive correlation between total research and development expenditure and the level of high-tech exports.

Recently, Panda and Sharma (2020) explore the technological specialisation of countries in different fields and its contribution to high-technology exports in developing countries, employing in their research revealed technological advantage (RTA) index and revealed symmetric technological advantage (RSTA) index. They find strong evidence that technological specialisation translates into high-technology export. Sepehrdoust et al. (2021) investigate the impact of scientific productivity on high-technology exports in selected developing countries during 1996–2017, reaching the conclusion that factors such economic risk, scientific productivity, financial risk and political risk have significant effects on the high-technology export. Drapkin et al. (2021) studied the determinants of high-tech exports in the Central and Eastern Europe countries, concluding that many factors stimulate the export growth in high-tech industries of the studied countries, such as the level of wages and resources prices, openness of the economy to foreign trade, tax rate, unemployment rate and the quality of human capital. Mulliqi (2021) examines the role of education in explaining the technology-intensive exports of 27 European countries, considering a comparative analysis of transition and non-transition economies, finding a positive and highly significant relationship between higher levels of education and technology-intensive exports. Özsoy et al. (2022) study the impact of digitalisation on exports of high-technology products using the ICT Development Index (IDI), concluding that in developing countries, IDI has a significant effect on export of high-tech products.

## Data and Methodology

Data for the analysis of the drivers of exports of technology-intensive manufactures come from the UNCTADstat database, the World Bank database and the OECD database. As indicated above, the panel contains data for all 35 OECD countries covering 15 years (2004 to 2018). However, data for the variables used are not available for all countries and all years; hence, an unbalanced panel is provided. The category of manufactures with high-technological content, according to the Lall (2000) classification, includes manufactures that have used high technology in their production with an important R&D content, that also require the use of skilled labour, that are able to adapt to an environment of continuous technological change on both the demand and the supply side. Moreover, this type of manufacturing requires production processes that can vary in the short term, where global value chains play a more important role than in manufactures with a lower technological content. High-tech manufacturing industries include aeronautics, pharmaceuticals, office machinery, accounting and computing, communications equipment and medical, precision and optical instruments, among others.

In line with the literature, two alternative measures have been considered to measure the importance of high-technology content manufacturing exports:

### High-tech Manufacturing Exports

Authors such as Seyoum (2004), Gökmen and Turen (2013), and Kabaklarli et al. (2017), have used this variable in the analysis of technology-intensive exports drivers. Data are expressed in thousands of current dollars and are sourced from UNCTADstat database.

### High-tech Manufacturing Exports as a Share of Total Employment

Tebaldi (2011) uses this variable in his study. This variable gives the value of exports measured in terms of labour endowment in the economy. Data for exports of high-content manufactures are from the UNCTADstat database and are expressed in thousands of current dollars. Labour endowment data are collected from the OECD database and are expressed in thousands of workers.

Considering these two measures and the data panel framework, the following model has been proposed:

$$\begin{aligned}{\mathrm{log}(XHIGH)}_{it}=&\; {\beta }_{0}+ {\beta }_{1}{\mathrm{log}\left(GFCF\right)}_{it}+{\beta }_{2}\mathrm{log}\left({LAND}_{it}\right)\\&+ {\beta }_{3}\mathrm{log}\left({UNIVERSITY}_{it}\right)+ {\beta }_{4}{\mathrm{log}\left(R\&D\right)}_{it}\\&+ {\beta }_{5}{FDI}_{it}+ {\beta }_{6}{IMPORTS}_{it}+{\beta }_{7}\mathrm{log}({POP}_{it})\\&+{\beta }_{8}{INSTITUTIONAL}_{it}+{\beta }_{9}{EUROPE}_{it}+\alpha t+{\nu }_{i }+ {\varepsilon }_{it}\end{aligned}$$

where

“XHIGH” captures each of the two measures of technology-intensive exports considered.

“GFCF” represents gross fixed capital formation over total employment and captures a country’s factor endowments in physical capital and labour. Gross fixed capital formation is a proxy variable for physical capital and has been frequently used in the economic literature (Zhu & Fu, 2013; Pierzak, 2015; Gourdon, 2009). The data for gross fixed capital formation is obtained from the World Bank database and measured in current dollars, while the data for total employment are from the OECD database.

“LAND” represents the land area per capita. Data are obtained from the World Bank’s open database.

“UNIVERSITY” measures the percentage of university graduates relative to the population group. It is a proxy variable for human capital frequently used in the economic literature (Zhu & Fu, 2013; Henn et al., 2014; Blanchard & Olney, 2017). The underlying importance of human capital in the OECD technology classification lies in the relevance of this variable, since as the technological sophistication of manufacturing increases, it is necessary to incorporate human capital with higher technological skills into production. These skills are characterised by an important ability to adapt to technological changes and new production processes in a short space of time. University graduates are characterised by a greater ability to adapt to changes in new production processes.

“R&D” measures R&D expenditure in terms of GDP in current dollars. The source is the World Bank database. “R&D” is a proxy for endogenous innovation and knowledge creation and has been frequently used in the literature (Zhu & Fu, 2013; Bravo-Ortega et al., 2014; Bournakis & Tsoukis, 2016). Additionally, extensive research has been carried out on the role that innovation plays in the export capacity of countries (Lall, 2001; Nassimbeni, 2001; Sandu & Ciocanel, 2014).

“FDI” is the stock of inward FDI in terms of GDP, measured in current dollars. The source is the UNCTADstat database. It is a proxy variable for exogenous innovation and is frequently used in the literature on export drivers (Zhu & Fu, 2013; Wacker et al., 2016). The rationale for the analysis of this variable is that the spillovers of FDI into technology-intensive exports derive from the advanced technology and management skills that multinational firms carry with them, along with access to global, regional and especially home country markets (Potterie & Lichtenberg, 2001; Zhang, 2006).

“IMPORTS” are imports of high-tech manufactures as a share of GDP, measured in thousands of current dollars. The source is the UNCTADstat database. “IMPORTS” is as a proxy variable for exogenous innovation and widely used in the literature (Xu & Chiang; 2005; Zhu & Fu, 2013).

“INSTITUTIONAL” measures the quality of national governance and regulation and captures the ability of governments to design and implement policies that promote private sector development. It is measured by a percentile ranging from 0 to 100, the higher the percentile the better the perception of the regulation designed and implemented. This variable is designed by the World Bank and is obtained from the World Bank’s development indicators database. These types of institutional quality variables are commonly used in the literature (Zhu & Fu, 2013; Gani & Prasad, 2006).

“POP” is the population in thousands of people and measures the size of the exporting economy. Data are obtained from the World Bank’s open database. Population has been a commonly used variable in literature (Zhu & Fu, 2013; Kalyvitis, 2015).

“EUROPE” is a binary variable that takes value 1 if the country is part of the European Union and takes value 0 otherwise.

“$\alpha t$” is a common time effect, represented in this case by a trend.

“${\nu }_{i}$” is a random effect linked to each country.

“${\varepsilon }_{it}$” is a random disturbance with the usual properties.

## Results

Table 1 shows the results obtained for the estimations of the two models proposed in the previous section to explain the drivers of exports of high-technology content manufactures. For each model, results obtained are displayed using generalised least squares (GLS) and generalised method of moments (GMM). In the context of GMM, to test the joint validity of all instruments and the likelihood of overidentifying restrictions the *J*-statistic is employed, being consistent in the presence of heteroscedasticity and inter-cluster correlation. Moreover, as mentioned in the previous section, to capture joint time effects, it has been decided to incorporate a time trend rather than time fixed effects in the model. This allows the possible time trend evolution of the different measures of high-tech manufacturing exports to be captured in a simple way. This modelling, more in line with time series models that capture the existence of inertial and trend components, seems to us to be more appropriate for a panel of countries with 15 years than incorporating dummy variables for years without any consideration of the possible existence of trend behaviour.

**Table 1:** Models for determining high-tech manufacturing exports

	Measures of technology-intensive exports			
	High-tech manufactured exports^(a)^		High-tech manufactured exports/labour^(a)^	
	(I)		(II)	
	GLS	GMM	GLS	GMM
Constant	−0.299 (0.489)	−6.248^(***)^ (4.937)	−5.954^(***)^ (0.522)	−8.367^(***)^ (2.010)
GFCF^(a)^	0.495^(***)^ (0.064)	0.313^(*)^ (0.168)	0.461^(***)^ (0.063)	0.093 (0.218)
LAND^(a)^	−0.049 (0.045)	0.027 (0.070)	−0.060^(*)^ (0.036)	−0.069 (0.064)
UNIVERSITY^(a)^	0.470^(***)^ (0.080)	1.700 (1.275)	0.409^(***)^ (0.086)	1.080^(*)^ (0.648)
R&D^(a)^	0.180^(***)^ (0.017)	0.022^(***)^ (0.065)	0.171^(***)^ (0.019)	0.244^(***)^ (0.068)
FDI ratio	0.0006^(*)^ (0.00035)	0.015^(*)^ (0.008)	0.0006^(*)^ (0.00038)	0.010^(***)^ (0.004)
IMPORTS	0.091^(***)^ (0.007)	0.065^(**)^ (0.027)	0.094^(***)^ (0.007)	0.065^(***)^ (0.013)
POPULATION^(a)^	1.006^(***)^ (0.045)	1.287^(***)^ (0.185)	0.016 (0.048)	0.214^(***)^ (0.080)
INSTITUTIONAL	−0.00001 (0.0008)	0.0005 (0.001)	−0.0005 (0.0006)	0.0155 (0.0167)
EUROPE	0.488^(***)^ (0.060)	0.821^(**)^ (0.278)	0.447^(***)^ (0.081)	0.716^(***)^ (0.168)
Time trend	−0.006^(***)^ (0.002)	−0.066 (0.047)	−0.006^(***)^ (0.002)	−0.043^(**)^ (0.022)
Standard error	0.163	0.351	0.163	0.289
Global significance test/*p*-value	6562.359 (0.000)	16,426.80 (0.000)	1041.242 (0.000)	405.613 (0.000)
J-statistic/*p*-value	-	0.017 (0.895)	-	5.284 (0.152)
Sample size	471	443	471	443

^(*)^Significant at 10%; ^(**)^Significant at 5%; ^(***)^Significant at 1%

^(a)^Log-transformed variable. Robust standard errors in parentheses

As can be seen, the results obtained when estimating by GLS and GMM are quite similar. We focus on interpreting the GMM results in which we control for possible country-specific heteroskedasticity problems. Columns I and II of Table 1 present the results obtained for the model of total exports of high-technology content manufactures and for the model of total exports over employment. It is worth noting that:

1. The expected signs are obtained for all the variables except for institutional quality, for which a negative sign is obtained, although it is not statistically significant at the usual levels of significance. In any case, the non-significance of this variable is an expected result given that the OECD countries considered in the analysis are countries with a good level of institutional performance
2. The GFCF effect has a positive sign, suggesting that an increase in capital investment in terms of employment is linked both to higher total levels of high-tech manufacturing exports for OECD countries and to such exports in terms of employment. Tebaldi (2011) finds that GFCF does not have a statistically significant effect at 5%. These differences are possibly since Tebaldi (2011) considers a broad set of countries including both high- and low-income countries.
3. Positive sign of UNIVERSITY, which approximates the average level of human capital across economies, indicates that human capital has a positive impact on technology-intensive exports. This result is fully in line with Tebaldi (2011) and Seyoum (2004).
4. In line Braunerhjelm and Thulin (2008), R&D has a positive effect on high-tech manufacturing exports both in absolute terms and in terms of employment. The positive effect of R&D and UNIVERSITY shows that knowledge creation activities have a positive impact on high-tech manufacturing exports.
5. As obtained by Tebaldi (2011) and Seyoum (20,004), the results show that FDI plays a positive role in the exports of high-tech manufactures. This outcome, along with the fact that imports of high-tech manufactures, as a source of exogenous innovation, affect positively the capacity to export high-tech manufactures, shows that economic openness is a driver in the increase of such exports. Moreover, the role of imports of high-tech manufactures in stimulating exports of such manufactures may also be related to the existence of important international value chains. Thus, imports of technology-intensive manufactures are an intrinsically interlinked in the same export value chain.
6. POP has a positive and statistically significant effect on high-tech exports both in absolute terms and in terms of employment. This effect suggests that the size of the economy, and hence, the size of the market in which domestic industries operate positively affects high-tech exports.
7. In both models, a statistically significant decreasing time trend effect is estimated. Thus, for both total exports and the share of exports in employment, a negative trend growth of around 6% per year is estimated.

## Discussion

In the previous analysis, we have shown that for the period under study, gross fixed capital formation in terms of employment is a driver of both variables, the total volume of technology-intensive manufacturing exports and the volume of technology-intensive manufacturing exports in terms of total employment. There is a broad consensus on the positive effects that gross fixed capital formation, such as investment in infrastructure, has for the economies of advanced countries at the macroeconomic level, mainly because of its impact on supply side capacity (Acemoglu & Robinson, 2015; Barro, 1991; Bassanini & Scarpetta, 2003; De la Fuente-Mella et al., 2020; Sala-i-Martin et al., 2004). In contrast, the effect of gross fixed capital formation on the ability of countries to export products with higher technological intensity has been less thoroughly researched.

At present, most economies in advanced and developing countries are suffering a major decline due to the COVID-19 pandemic and the war in Ukraine. As a result, many OECD countries have designed measures to deal with the devastating consequences of the pandemic. It is worth remembering that fixed capital investment is also an important counter-cyclical tool (International Monetary Fund, 2014). However, the use of this tool can lead to significant fiscal imbalances at a time when the public finances of many countries are severely deteriorated due, among other things, to the implementation of aid schemes to alleviate the effects of the pandemic on the labour market and to the implementation of compensatory measures for the compulsory shutdown of companies due to the ongoing confinements for health reasons. To mitigate the abovementioned disruptive effects on public finances, public–private partnerships (PPP) should be explored.

In our analysis, population has a positive and statistically significant effect on technology-intensive exports both in absolute terms and in terms of employment. This effect indicates that the size of the economy, and hence, the market size in which domestic industries operate positively affects high-tech exports.

On human capital, we can conclude from the data obtained that the percentage of university students in terms of the population group in OECD countries is a positive driver of the total value of exported manufactures with high-technological content and a driver of the value of this type of manufactures in terms of labour. From the economic policy perspective, measures aimed at improving and encouraging higher education could result in an increase in the volume of exports with a higher technological content and in a higher specialisation of the exporting sectors of manufactures with a higher technological content. A wide range of economic and social policy measures that positively impact on the enhancement of human capital are feasible, ranging from increasing and better rationalising budget spending on higher education, increasing flexibility in the organisation of higher education institutions, promoting and fostering international mobility of faculty and students, improving incentives for students to complete their higher education studies by improving scholarship and loan systems, etc. (OECD, 2017).

A further important finding is that endogenous innovation, and more specifically R&D investment in terms of GDP, positively affects both the total value of high-tech manufactures exported by OECD countries and the total value of high-tech manufactures in terms of total employment. These findings have important policy implications since measures that lead to an increase in the R&D/GDP ratio could produce positive effects on the value of high-tech manufacturing exports in OECD countries. Potential policy measures to increase this ratio include promoting international cooperation among academics, as it is linked to research excellence (OECD, 2019); increasing the number of PhDs, as it is linked to higher R&D intensity (OECD, 2019); promoting business-university collaboration in the knowledge creation and innovation process; encouraging the creation and consolidation of technology parks; encouraging firms to invest more by promoting access to finance, access to venture capital and fostering angel investor firms (OECD, 2015); favouring strong and efficient systems regarding knowledge creation and diffusion (OECD, 2015); implementing innovation policies that foster the overcoming barriers to innovation processes, such as the obstacles faced by large value chains in their innovation processes (OECD, 2015); favouring the involvement of SMEs in innovation processes, etc.

In the literature, we find empirical studies that positively link the size of OECD countries’ economies with exports (Wang et al., 2010); in our studies, we find evidence that the size of the economy plays a certain role in determining the total value of export flows of manufactured goods with high-technological content, on these total flows in terms of employment and on productive specialisation in manufactures with higher technological content.

Exogenous innovation, proxied in our analysis by the stock of inward FDI and imports of technology-intensive manufactures, also plays a positive role in determining the total value of exported technology-intensive manufactures and in determining the value of exports of technology-intensive manufactures in terms of employment. Likewise, the stock of inward FDI and imports is drivers of technological specialisation, contribute to increasing the share of exported high-technology content manufactures in total exported manufactures and contribute to greater technological content, improving comparative advantages in those manufactures with a higher technological content. Based on these findings, policies that favour greater trade openness in the import of products with a higher technology content and those that remove barriers in the import processes of large value chains (OECD, 2018) could favour a higher technology content of manufactured exports. Likewise, those that encourage capital inflows through FDI, with improvements in tax incentives, better profit repatriation processes, fostering international labour mobility, etc., could have a positive effect on the volume of exports with a high-technological content, as well as on the specialisation and technological sophistication of OECD countries.

## Conclusions

Empirical studies analysing the determinants that lead some countries to export more than others are quite extensive in the literature. However, research delving deeper into the drivers of high-technology content manufacturing exports is scarce. This article has attempted to contribute to filling part of this gap by analysing key variables as likely drivers for the improvement of the technology content of manufacturing exports in OECD countries. In addition, this paper sets out a menu of policy measures that would improve the technology mix of exports from the countries in the study.

Through the econometric analysis of the proposed model, we have determined the relationship between the endogenous variables, high-tech manufacturing exports and high-tech manufacturing exports as a share of total employment and a number of exogenous variables such as gross fixed capital formation on total employment, the land area per capita, the percentage of university graduates relative to the population group, R&D expenditure in terms of GDP, the stock of inward foreign direct investment in terms of GDP, imports of high-tech manufactures as a share of GDP, the quality of national governance and regulation, the population and EU membership. Model estimations have been carried out using GMM and GLS estimators.

Among the main results of the empirical analysis, we find that the physical capital variable, the human capital variable, the innovation, the foreign direct investment, imports of high-tech manufactures, country size in terms of population and the EU memebership play a significant and positive role in determining exports of technology-intensive manufactures.

Although the results obtained are of significant relevance, some limitations of the study should be pointed out, and these will be the basis of future lines of research. The set of countries considered in the analysis, insofar as they are all members of the OECD, have the characteristic of being quite homogenous from an institutional and economic development point of view. But, it would be interesting to analyse with current data to what extent these results are valid for other sets of countries. On the other hand, the outbreak of the COVID-19 pandemic and the war in Ukraine seem to have changed the prevailing context of international trade relations. It would therefore be interesting to analyse how these shocks may have affected the results obtained.

## Data Availability

Data for the analysis of the drivers of exports of technology-intensive manufactures come from the UNCTADstat database, the World Bank database, and the OECD database: https://unctadstat.unctad.org/EN/, https://databank.worldbank.org/, https://stats.oecd.org/.
